# A dual fusion recognition model for sleep posture based on air mattress pressure detection

**DOI:** 10.1038/s41598-024-61267-0

**Published:** 2024-05-15

**Authors:** Zebo Li, Yipeng Zhou, Guoping Zhou

**Affiliations:** College of Information Science and Technology & College of Artificial Intelligence, Nanjing Forestry University, No.159 Longpan Road, Nanjing, 210037 Jiangsu People’s Republic of China

**Keywords:** Sleep posture recognition, Ensemble learning, Spearman correlation coefficient analysis, AdaBoost-SVM, Mattress pressure detection, Biomedical engineering, Electrical and electronic engineering

## Abstract

In order to solve the difficult portability problem of traditional non-invasive sleeping posture recognition algorithms arising from the production cost and computational cost, this paper proposes a sleeping posture recognition model focusing on human body structural feature extraction and integration of feature space and algorithms based on a specific air-spring mattress structure, called SPR-DE (SPR-DE is the Sleep Posture Recognition-Data Ensemble acronym form). The model combines SMR (SMR stands for Principle of Spearman Maximal Relevance) with horizontal and vertical division based on the barometric pressure signals in the human body’s backbone region to reconstruct the raw pressure data into strongly correlated non-image features of the sleep postures in different parts and directions and construct the feature set. Finally, the recognit-ion of the two sleep postures is accomplished using the AdaBoost-SVM integrated classifier. SPR-DE is compared with the base and integrated classifiers to verify its performance. The experimental results show that the amount of significant features helps the algorithm to classify different sleeping patterns more accurately, and the f1 score of the SPR-DE model determined by the comparison experiments is 0.998, and the accuracy can reach 99.9%. Compared with other models, the accuracy is improved by 2.9% ~ 7.7%, and the f1-score is improved by 0.029 ~ 0.076. Therefore, it is concluded that the SMR feature extraction strategy in the SPR-DE model and the AdaBoost-SVM can achieve high accuracy and strong robustness in the task of sleep posture recognition in a small area, low-density air-pressure mattress, taking into account the comfort of the mattress structural design and the sleep posture recognition, integrated with the mattress adaptive adjustment system.

## Introduction

High-quality sleep is essential for human health^1^. The sleep process is also a process by which a person recovers energy, consolidates memories, and promotes physical health^2^. Sleep posture directly affects the quality and depth of sleep. Improper sleep posture may lead to body pain, breathing obstruction, or even long-term health problems, for example, sleeping on the back may exacerbate sleep apnea syndrome and lead to gastroesophageal reflux disease (GRED)^3^, and prolonged side-lying sleeping may lead to shoulder pain or exacerbate pre-existing shoulder problems^4^. Modern monitoring devices and apps, however, can track changes in sleep posture in real-time, analyze sleep patterns, and provide targeted recommendations and adjustments. For example, sleeping on the side can alleviate sleep apnea syndrome and reduce acid reflux. In the supine sleep posture if proper support is provided for the cervical spine, back, lumbar spine, and hips can relieve pressure or pain in the neck and lower back^5^. Proper sleep posture monitoring and adjustment can also help some specific groups of people. For example, during the postoperative recovery period according to the patient’s condition, some specific sleep posture change adjustment strategies are used by monitoring their sleep posture in real-time. For pregnant women, real-time monitoring of the sleep posture and making appropriate adjustments, adopting the fetal sleep posture, and providing appropriate support can relieve low back pain and ensure fetal health at the same time.

During sleep, it is advantageous to recognize sleep postures based on pressure perception in the main body regions. In particular, in terms of human biomechanics^6–8^, the shoulders, back, and hips serve as the main support points of the body^6^, and their positions and corresponding pressure distributions contribute to the curvature of the spine and the overall stability of the sleep posture^7,8^. Sleep physiology studies^9–12^ have shown that shoulder-hip pressures and spinal pressures can provide information about whether an individual maintains a sleep posture that reduces body stress and supports the natural curve of the spine^10,11^, which is critical for understanding sleep quality and preventing sleep-related disorders^12^. This has led to theoretical and practical research support for considering shoulder-hip eigenquantities and spine eigenquantities as key eigenquantities in sleep position recognition.

The key to solving these problems lies in proposing a feature extraction algorithm, and sleeping pattern recognition model that can target the integration of sleeping pattern recognition and mattress adaptive adjustment system for air mattress structure. The effective feature quantity is utilized to help the algorithm classify different sleeping patterns more accurately, thus improving the performance of the recognition system.

Specifically, the sleep posture recognition model (1) is accurate in its ability to analyze pressure distribution, and the model is able to accurately map the pressure distribution pattern of the human body on the mattress. This includes identifying the location and pressure magnitude of the body’s major pressure areas and the alignment of the spine. (2) Application of ergonomic principles to assist in feature extraction to ensure that the extracted features reflect the biomechanical and pressure distribution characteristics of the human body in different sleep postures. (3) Be highly sensitive to the spatial relationships between body parts and the dynamic properties of these relationships as they change with sleep posture, including the differences in pressure on the mattress from different parts of the body (4) Identify changes in pressure in different directions, and understand how these changes characterize different sleep postures and their impact on sleep comfort and support needs.

Combining the highly focused feature extraction capability of the model and the sensitivity to spatial relationships and variations, the research in this paper addresses the problem of sleep posture recognition in the context of air pressure mattress applications and proposes a dual-fusion recognition algorithm for sleep posture based on air pressure detection of mattresses-the SPR-DE algorithm.

The contribution of this paper has three main parts:

- (1) SMR (Principle of Spearman Maximal Relevance) feature extraction strategy is proposed, the subset of salient features obtained by SMR contains features related to sleep postures, and the non-image data can be used as input to the sleep posture recognition model. Among them, the horizontal and vertical division strategy focuses on obtaining characteristic quantities about the body’s main support areas (shoulders, hips) and spine status, which are key indicators for assessing sleep comfort and health. It is beneficial for the system to more accurately determine the sleep posture and the areas that need to be adjusted in order to provide the best support, such as adjusting firmness and matching pressure distribution for specific body parts; compared to image data, the barometric pressure data used for model input is easier to obtain from the built-in sensors of the mattress, which simplifies the system’s hardware requirements and reduces the intrusiveness of privacy; and the computational cost is lower for the non-image data, especially for the transversal and longitudinal division feature volumes, which can be used for the assessment of the sleep comfort and health. volumes, the lower computational cost enables faster data processing, which means that the system can be adjusted in real-time without compromising performance, improving the user experience. This model is easier to integrate into a variety of mattress systems due to the reduced data processing requirements and simplified hardware requirements (no need for complex image acquisition and processing equipment). This makes it easier to balance mattress comfort with the portability of sleep posture recognition in designing the sleep posture recognition system.
- (2) The horizontal and vertical division approach used in the SMR strategy reveals that the feature vectors of the shoulder region and hip region in the SBWH feature subset and the feature vectors related to the spine line in the LMR feature subset are the crucial features. Sleep posture changes can optimize pressure distribution across the body, reduce the risk of pain or injury from pressure concentrations, excessive stress, or strain, and maintain the body’s ability to maintain balance in both dynamic and static states. From an ergonomics and biomechanics point of view, shoulder-hip characteristic quantities (e.g., pressure distribution at the point of contact with the bed surface) provide important information for assessing whether a sleep posture is conducive to reducing pressure concentrations and improving blood circulation. The S-curve structure of the human spine, which includes anterior convexity of the cervical spine, posterior convexity of the thoracic spine, and anterior convexity of the lumbar spine, makes spinal characteristic quantities (e.g., pressure distributions of the spinal curves) critical for identifying healthy and unhealthy sleep postures. The characteristic quantities of the shoulder-hip and spine provided by this division extraction approach provide a basis for evaluating not only the effects of sleep postures on the stress distribution in various parts of the body but also the effects of sleep postures on maintaining stability and preventing over-twisting or over-extension. This in turn improves the accuracy of the model classification.
- (3) The classification model adopts the AdaBoost classification algorithm under the Boosting framework, which is used in combination with the weak classifier SVM to train the weak classifier iteratively, adjusting the sample weights according to the iterative errors in each round, and enhancing the learning of difficult-to-classify samples. When dealing with different types of feature information, the weights of individual features are adaptively adjusted; the strong ability to deal with nonlinear features in SVM is most critical to naturally differentiate the number of features in which divisions are used, and AdaBoost-SVM introduces a regularization term while increasing the complexity of the model, helping to control the overfitting, especially for the horizontal and vertical division features that contain a large amount of detailed information, which helps to improve the accuracy and generalization ability. This not only better matches the SMR feature extraction strategy, but also greatly improves the performance indexes of the classification model.

The remainder of the paper is organized as follows: Chapter 2 reviews related work, and Chapter 3 describes the materials and methods, including the division of the air bed and the introduction of the embedded system and the research. Chapter 4 describes the framework of the model, including data preprocessing, SMR feature extraction strategy, construction of feature subsets, and AdaBoost-SVM classification algorithm. Chapter 5 includes data preparation and experimental results, and finally, Chapter 6 gives a discussion and Chapter 7 gives conclusions and future work.

## Related work

The current sleep posture recognition and adjustment system is realized by different hardware and sensors, which not only increases the number of sensors and production cost, but also increases the complexity of system design and the difficulty of data computation. The sleep posture recognition model for air beds proposed in^13^ still uses image data as input, which is computationally intensive and not very portable. In contrast, the design of air springs^14^, as well as the design of the air bed structure^15^ makes the integration of sleep posture recognition and mattress adaptive adjustment system possible. Currently, the mainstream detection methods for invasive^16^ and non-invasive^17^ sleep posture recognition are categorized into three main types: wearable device-based detection methods, vision-based detection methods, and pressure detection-based methods.

All of these sleep posture recognition techniques aim to extract features and classify sleep postures from the relevant data collected by hardware devices.

Wearable device-based monitoring methods sense limb movements via accelerometers^18,19^ and typically employ wrist-worn sleep trackers to collect sleep data. Worn on the wrist by the subject, although they are powerful devices with built-in sensors, they are both expensive and invasive and can cause discomfort to the user.

Vision-based monitoring methods use depth sensors^20^ or fusion cameras^21^ ^22^. Sleep posture recognition is achieved through global features from depth images. Although vision-based methods are characterized by low cost and easy maintenance, they require high light conditions without occlusion and can involve issues of user privacy. In the past these methods, by using bone detection and differential information or by wireless solutions that depend on special devices, but it is difficult to achieve accurate pose recognition because the use of bedding and the shooting conditions have a significant effect on the accuracy. In recent years, methods using radar sensors have also been developed^23^. Examples include traditional detection techniques (TDT)^24^, traditional machine learning (TML)^25,26^ , and deep neural networks (DNN)^27,28^ . Although more stable, the sleep conversion algorithm also has the limitation of lower accuracy.

Distinguishing from the above methods, pressure-based detection methods are relatively more comfortable and natural, which usually use pressure pads embedded with pressure sensors to collect data and machine learning for sleep posture recognition. The number of sensors used in these methods varies^29^, four sensors around the bed and the recognition accuracy is related to the position of the person. Pressure sensors in^30^ are only 60 but need to be combined with a vision approach. Both of these methods suffer from poor recognition accuracy due to the low density of pressure sensors. Conversely, there have been studies that have used a large number of pressure sensors to obtain more localized details of the pressure distribution in the lumbar and hip^31^, using 1728 pressure sensors to localize the human limbs and 2048 in^32^. The pressure pads used in these methods are very large and require the use of a very large number of pressure sensors, leading to high computational complexity in the application of the system, and some of the sensors are expensive to produce, all of which create difficulties in the portability of the system. Kim placed a smart pad consisting of 128 FSR sensors between the mattress and the bed sheet for pressure measurement and proposed a sleep posture recognition algorithm based on the tier-1 model that uses only the three main parts of the upper body, shoulders, and hips to determine the sleep posture^33^. The average recognition accuracy was 87.9%. Although it was able to accurately recognize sleep postures based on the main parts of the body using a small number of FSR sensors, the recognition time was around 1 min. Matar et al. used a hardware implementation of a 27 × 6 FSR sensor array^34^, which converts the collected pressure data into image data and combines it with an ANN to perform classification, the sleep posture recognition system was able to reduce the data storage requirements and computation, and the The recognition accuracy can reach 97.6%. However, the processing of sleep posture recognition is too slow. Hu proposed a smart bed sheet based on an array of 1024 pressure sensors composed of conductive fabrics and wires^35^, which uses digital-to-analog conversion for data collection and CNN to recognize the sleep posture, and the system recognition accuracy can reach 91.24%, and the real-time processing speed can reach 434us/frame. Although these methods reduce the computational complexity, and production cost when the system is applied; however, these smart mattresses themselves can only be enhanced in recognizing sleep postures, and their design structure cannot be integrated with recognition and adjustment.

Second, pressure-based detection methods usually need to first convert pressure data into pressure images and perform feature extraction^20^. extracted the hidden sleeping features in ROI pressure images by CNN convolutional neural network^21^. Extracted sleeping features from flexible pressure sensor array data via ResNet’s algorithmic framework^22^. Optimal separation of the maximum boundary hyperplane is achieved by calculating the Euclidean distance between features in the high-dimensional space of RSS trajectories via SVM and K-nearest neighbor to extract the optimal features in the embodied RSS trajectories^23^. Extract the features of different parts of the pressure through the coding layer of RNN and CNN-based coding layer^24^. extracted features by combining weighted 2D shapes of pressure shapes with EMD and Euclidean distance matching. These methods have the following drawbacks: 1. The feature inputs used are relatively homogeneous and mostly based on local features and personalized training, 2. The pressure map metadata used needs to be preprocessed in a way that some valuable features are omitted.

The accuracy of sleep posture recognition based on pressure detection is also related to the selection of classification algorithms^25^. found that the recognition accuracy of KNN in static sleep postures can reach 98% and is not easily affected by changes in feature space^26^. utilized SVM binary classifier can reach 93.6% accuracy and can avoid overfitting problems. However, when the dataset changes, the accuracy of the SVM classifier has a wide range of changes^27^. used a Bayesian classifier to estimate the likelihood of continuous poses, which has the highest classification accuracy of 91.5% when the Bayesian probability coefficient reaches 0.7, and explored the possibility of a small number of sensors to achieve the recognition of sleep postures, eliminating the weighting effect and the bias between different sensor types. The classification algorithms used in these methods, while capable of achieving high recognition accuracy on the training set, have poor generalization performance and a wide range of variation in sleep posture recognition accuracy when the dataset contains noise and outliers.

Although many studies have made important theoretical progress^36–43^. However, there are still some challenges that need to be addressed in the field of integrating sleep posture recognition and adjustment in home mattresses, (1) Due to the information redundancy of the data and the ambiguity of the feature space, the pressure image, when used as a model input, does not guarantee an accurate sleep posture classification result while reducing the number of pressure sensors^36–41^. (2) The current sleep posture recognition model only obtains the key features of sleep posture recognition from pressure data, but not based on the global starting point that the sleep posture recognition and the adaptive adjustment system of the mattress are closely synergized, which requires the algorithm to be able to provide the number of features that can accurately guide the mattress on how to adjust to a specific sleep posture. (3) For air spring mattresses, a suitable sleep posture recognition model is needed to process the air pressure sensor data and make accurate sleep posture recognition. Therefore, this study aims to propose a sleep posture recognition model based on air-pressure mattresses that feature the human trunk region.

## Materials and methods

### Air pressure mattress division

**Figure 1:**
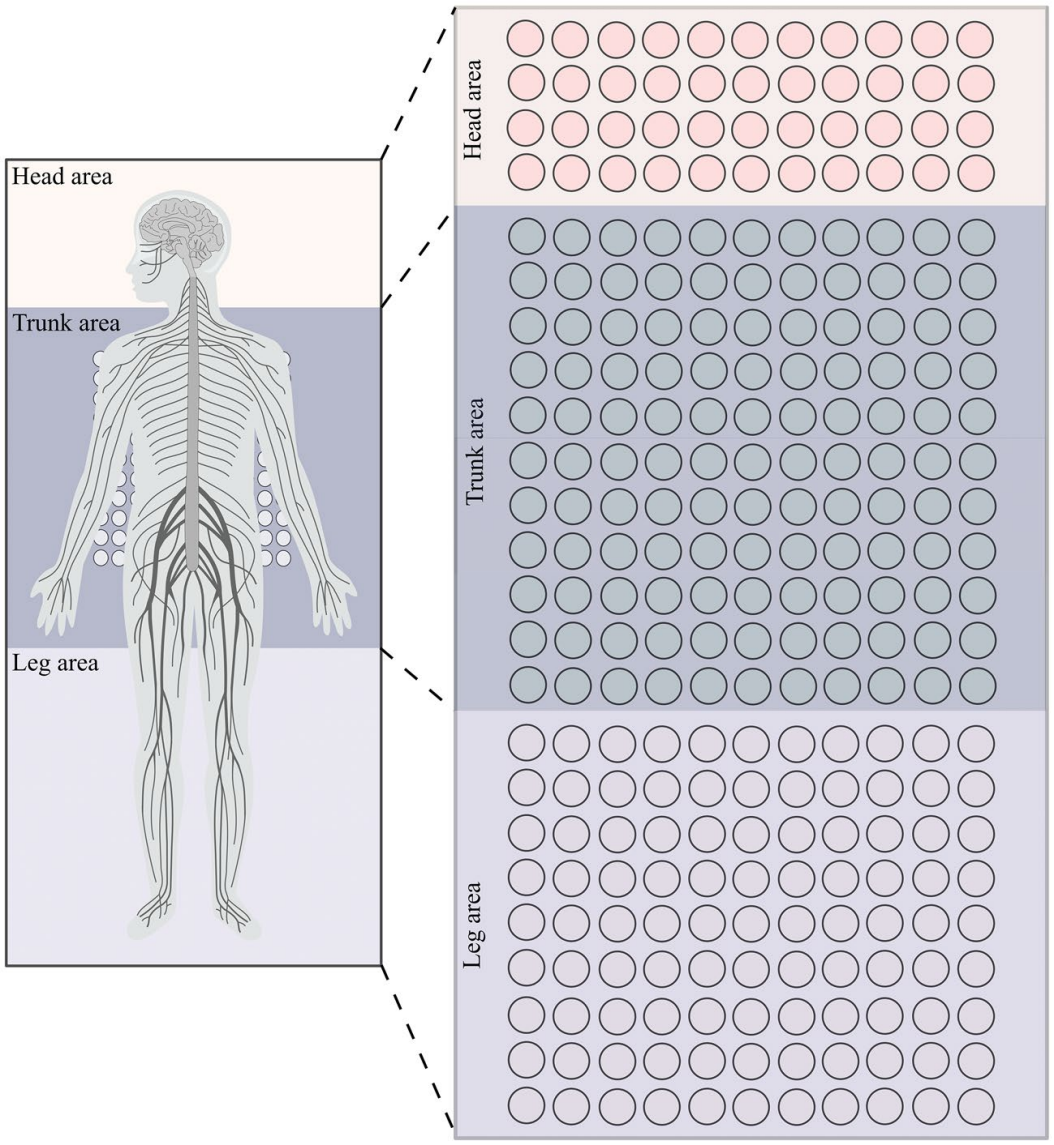
Diagram of mattress partitioning.

Considering the need to more accurately match the support needs of different parts of the human body with a small number of sensors, to improve the pressure distribution around the spine on the mattress, and to promote the retention of the natural curves of the spine, the air mattresses in this study are divided into the head, torso, legs and feet, and based on a combination of the principles of human ergonomics and the need for sleep comfort, as well as the accuracy and sensitivity of the recognition of the sleep postures. Figure 1 demonstrates the mattress division diagram. The head, although light in weight, requires proper support to maintain the comfort and health of the cervical spine. The torso section contains important organs and major body mass, so a larger area is allocated to provide adequate support and reduce the pressure on the back and lumbar region; the legs and feet, although they also need support requirements are not as high as the torso section, so the allocation ratio is slightly lower than that of the torso. While the larger trunk area reflects the influence of the center weight portion of the body on the mattress, the head legs, and feet provide additional information about changes in body posture. Therefore, the specific zoning of the air mattress is $4\times 11$ for the head, $11\times 11$ for the trunk, and $9\times 11$ for the legs and feet, aiming to provide optimal support and comfort for the sleeper, bringing a more comfortable and healthy sleeping experience, and enabling the designer to provide more precise and detailed data on sleep postures, improving the accuracy of sleep posture recognition.

### Embedded system structure

**Figure 2:**
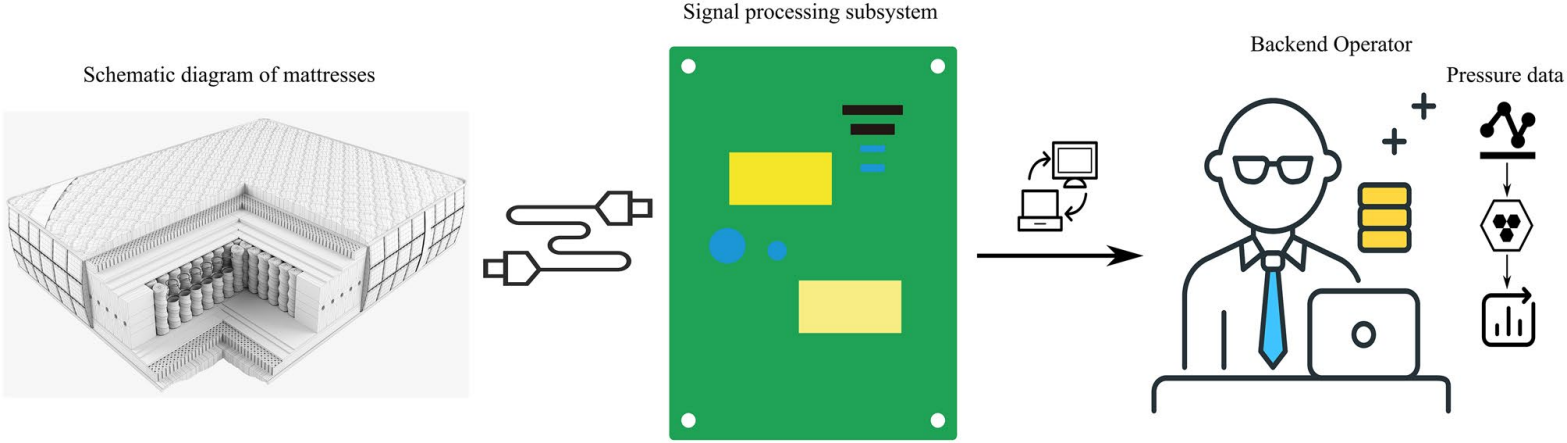
SPR-DE-based data acquisition system for sleep posture recognition.

Figure 2 demonstrates the SPR-DE-based data acquisition system for sleep posture recognition. The system consists of three components: an array of $11\times 24$ barometric pressure sensors modified by air springs, a data sampling unit, and a host computer. A master–slave control method was used. Other sensors on the circuit board can measure the air pressure in each branch when the corresponding solenoid valve is on. The pressure signal is collected by the air pressure sensors and sent back to the central control unit, which accepts and processes the signal to control the air pump and solenoid valve.

**Figure 3:**
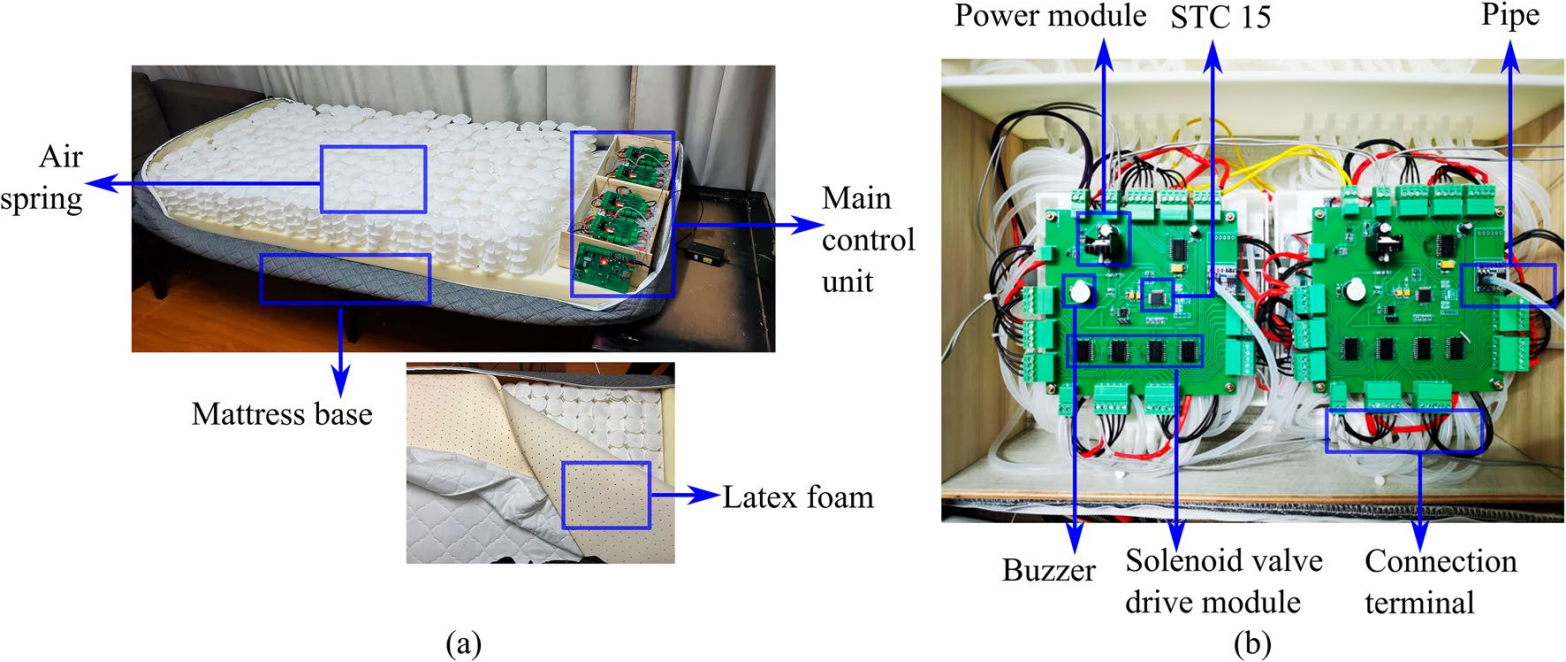
Physical map of the air spring mattress and embedded system (**a**) Air spring mattress (**b**) subordinative control board.

Figure 3 shows the composition of the air mattress bed and the hardware circuit version, the mattress system control box placed at the end of the mattress. In the upper layer of air springs select latex mattress, in order to ensure that the air tube can be smooth and will not be extruded under the circumstances of deformation, the selection of the diameter of 3 mm air tube, all air springs air circuit are connected to the air tube from the air springs below the sponge layer through the control box on the shunt interface connected to the control box.

In order to reduce the number of sensors and solenoid valves, the 121 air springs in the trunk are all independent airlines, and the rest are connected in series. The air spring air paths are connected to the shunt through solenoid valves, and each shunt is equipped with an air pump and air pressure sensor. Since all air paths are connected to the shunt, the system only needs to open the corresponding solenoid valves when it collects air springs’ air pressure or carries out air pressure regulation. The core of the host board adopts the STM32F103RCT6 chip, and the core of the slave board adopts the STC15F2K60S2 chip. It mainly includes modules of air pressure acquisition, data communication, data storage, air pump drive, and solenoid valve control.

**Figure 4:**
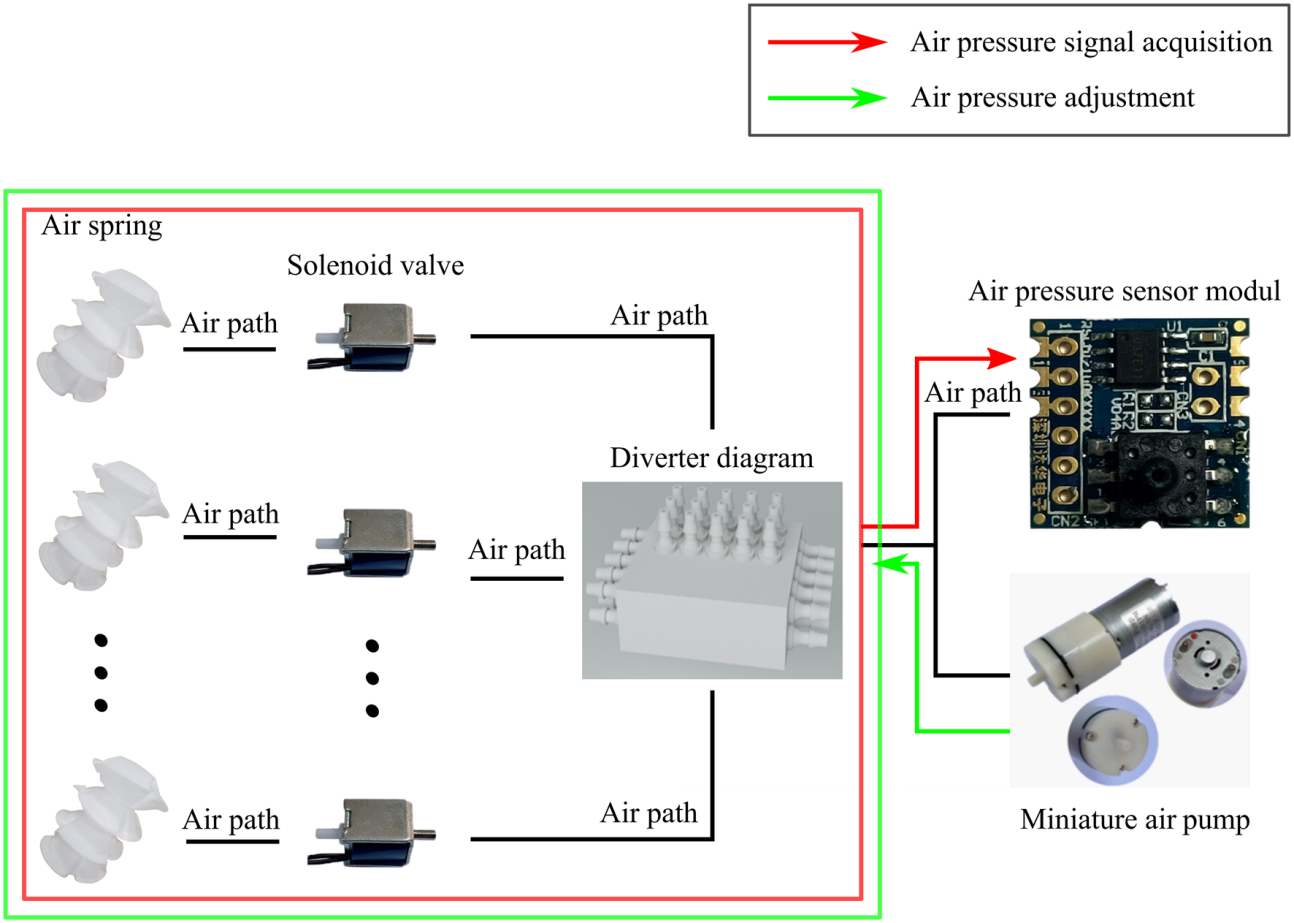
Gas springs retrofitted with gas pressure sensors.

Figure 4 gives the connection of the air spring’s with the solenoid valve, air pump, air pressure sensor, where the red color represents the air pressure data signal direction and the green signal represents the air pressure regulation signal direction. The air pressure sensor is model RSM17100KP100. The module contains differential amplification, automatic calibration, temperature compensation and other circuits, with the advantages of small size, fast response and strong anti-interference ability. Fa0520F miniature solenoid valves are selected, respectively, the straight-through valve and two-position three-way solenoid valve, which has the advantages of small size, low power consumption and so on. As the solenoid valve will produce heat when working for a long time, and only need to be opened when the air spring air pressure collection and regulation, so the selection of normally closed solenoid valves, open at the moment of need, normally closed at all times, reducing the overall power consumption of the mattress. Among them, the straight-through valve is used for the connection between the air spring and the shunt, and the two-position three-way valve is used for the quick deflation of the air spring. The air pump adopts ZR370-01PM miniature inflatable pump, which has the advantages of compact size, low noise, and can run continuously for 24 h.

### Research process

**Figure 5:**
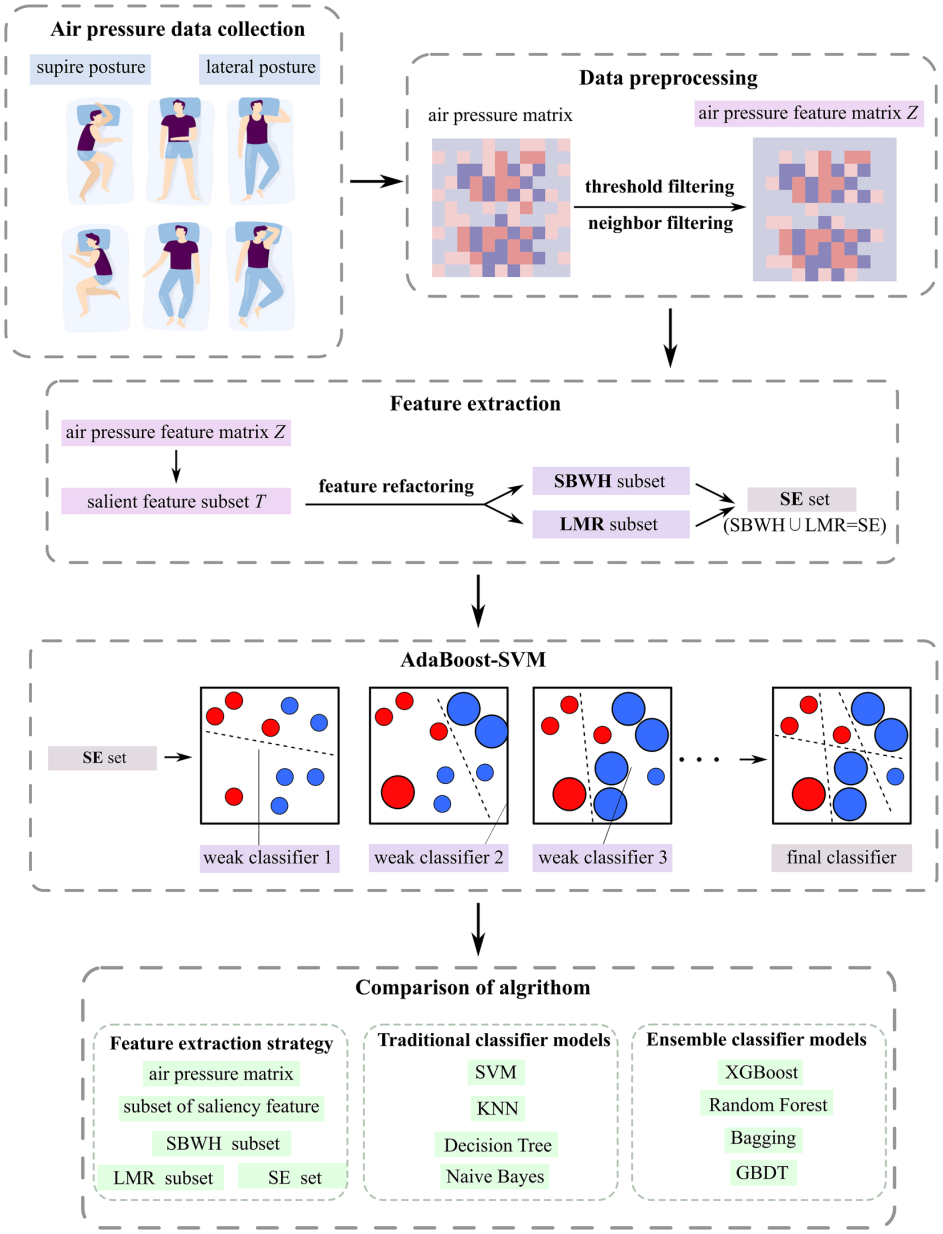
Schematic diagram of the SPR-DE study process.

Figure 5 shows the specific research flow of this paper, the research of the sleep recognition classification model SPR-DE includes four parts: data preprocessing, feature extraction, training of the integrated algorithm, and comparison and validation of the algorithm. Data preprocessing part of the threshold filtering and elimination of neighborhood noise effects to eliminate errors caused by the barometric pressure sensor as well as redundant information in the pressure data, where Z is the original barometric pressure data matrix after preprocessing to get *l* rows and *m* columns of the barometric pressure feature matrix. Pressure data and reconstructs the feature subset of the classification algorithm, and the effective feature subset can be more accurate for classification, in which the salient feature subset **T** is the distribution of the barometric pressure feature matrix **Z** in the low-dimensional space after sorting according to the principle of maximum rank correlation. The SBWH (shoulder, back, waist, hip) feature subset is the horizontal boundary feature subset obtained by reconstructing the salient feature subset T according to the distribution of the human torso structure and the SMR principle; the LMR feature subset is the longitudinal boundary feature subset obtained by reconstructing the salient feature subset T according to the different sleep postures and the SMR principle; and the SE feature set is the fusion of the SBWH feature subset with the LMR (Left, Middle, Right) feature subset, which is also the input of the classification algorithm. The integration algorithm adopts the algorithmic framework of Adaboost, with SVM as the base classifier, and finally, tenfold as well as LOOCV cross-validation are used to compare different classification algorithms as well as the integration framework, to validate the performance capability of the SPR-DE classification model for sleep posture recognition.

### Ethics approval and consent to participate

This study and included experimental procedures were approved by the committee of Nanjing Forestry University. All experiments were conducted in strict accordance with the institutional guidelines and regulations for care. All experimental protocols in this study were approved by the ethics committee of Nanjing Forestry University. We certify that the study was performed in accordance with the 1964 declaration of HELSINKI and later amendments. Written informed consent was obtained from all the participants prior to the enrollment of this study.

## Sleep posture recognition model

### Data and preprocessing

In the data preprocessing stage, to reduce the noise due to occasional malfunctioning of the barometric sensor. Threshold Filtering is used to reduce the system error:

1 $$p_{i,j} = \left\{ {\begin{array}{*{20}c} {\begin{array}{*{20}c} {p_{i,j} } & {p_{i,j} \ge Thre} \\ \end{array} } \\ {\begin{array}{*{20}c} 0 & {{\text{otherwise}}} \\ \end{array} } \\ \end{array} } \right.$$

2 $$Thre = \frac{{\mathop \sum \nolimits_{i = 1}^{11} \mathop \sum \nolimits_{j = 1}^{11} p_{i,j} }}{121}$$

where ${p}_{i,j}$ represent original barometric pressure sensor data, $1\le i\le 11$, $1\le j\le 11$.

Meanwhile, when a subject lies down, the air spring in contact with the body is squeezed, but some of the air spring that is not in contact with the body also shifts its position. Neighborhood filtering is applied to remove redundant information:

3 $$p_{i,j} = \left\{ {\begin{array}{*{20}c} {\begin{array}{*{20}c} {p_{i,j} } & {p_{i,j} \ge NThre} \\ \end{array} } \\ {\begin{array}{*{20}c} 0 & {{\text{otherwise}}} \\ \end{array} } \\ \end{array} } \right.$$

4 $$NThre = \frac{3}{4}*\min \left\{ {f_{i} } \right\}$$

where ${f}_{i}$ is the local maxima in each row of air pressure data.

**Figure 6:**
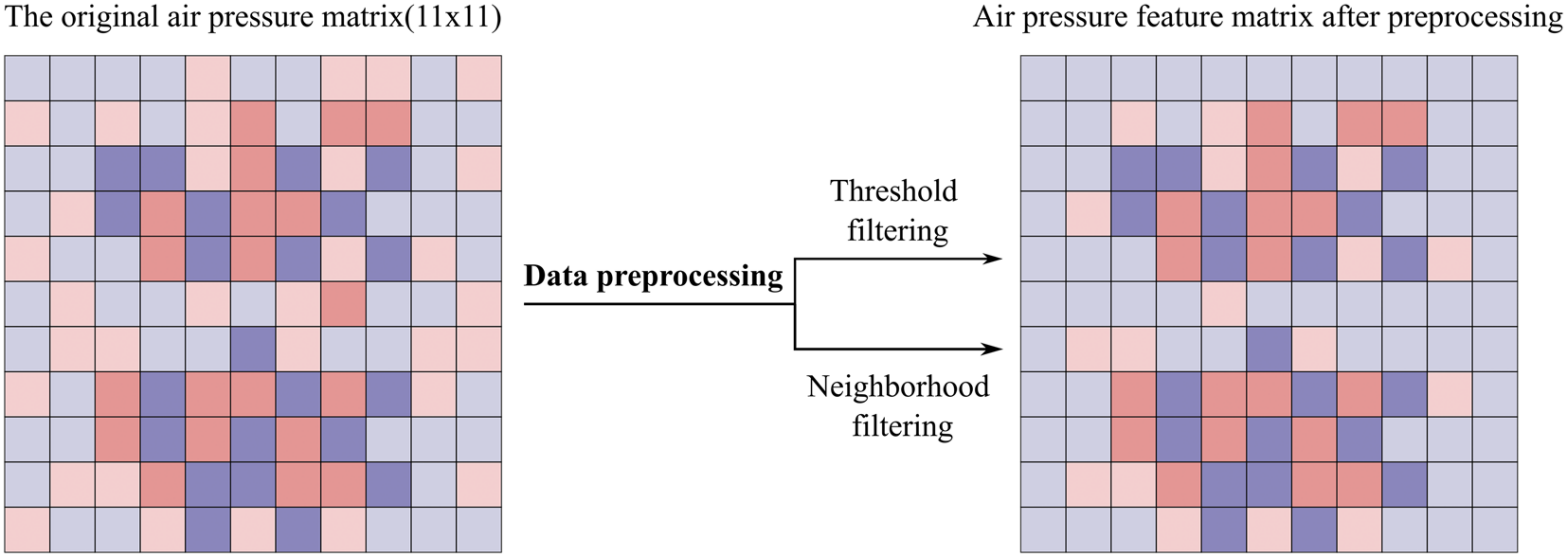
Schematic diagram of air pressure data before and after preprocessing.

Figure 6 shows the pressure data before and after preprocessing in the supine posture of the subject. After removing some redundant information, the information presented in the feature space of the pressure data is clearer, which is conducive to more accurate feature extraction.

### Feature extraction

Some pressure values of each part of the mattress vary depending on the structure of the human body and the sleep posture. 121 feature quantities in each sample are inconsistent with the relevance of sleep posture recognition. The feature dimensions are single, and the information is redundant. Before the classification training for sleep posture recognition, feature dimensionality reduction is needed for the air pressure feature space after preprocessing.

**Figure 7:**
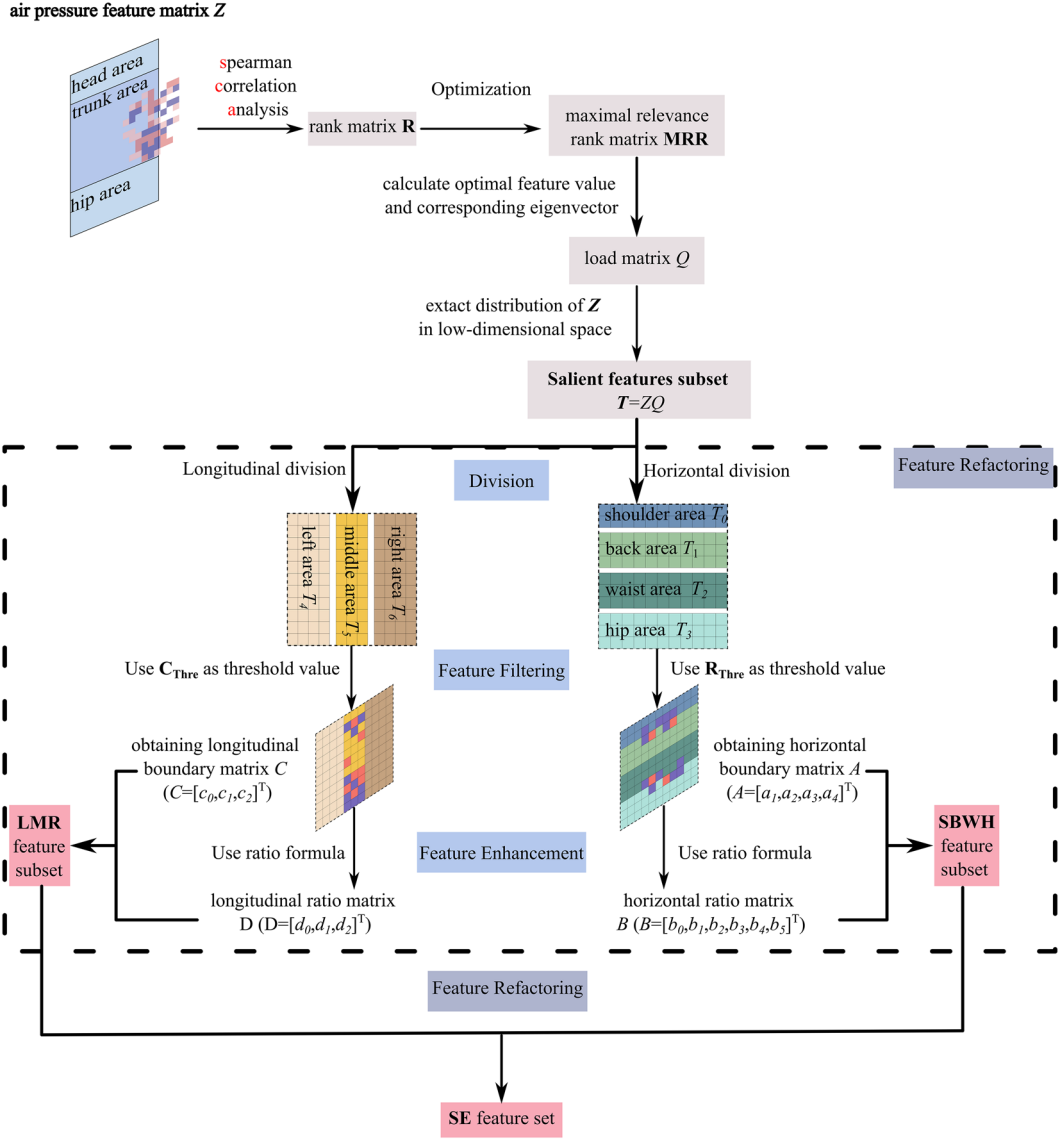
Flowchart of feature extraction.

Figure 7 shows the flowchart of the feature extraction strategy. Based on the human body structure and the different sleep postures. It is clear that the SMR will be explained in detail later in this section. With horizontal and vertical division, the SBWH (Shoulder, Back, Waist, Hip) feature subset and the LMR (Left, Middle, Right) feature subset are constructed, which finally are merged and fused into the SE (Sleep Ensemble) feature set, used as the classifier’s input.

The SBWH feature subset and the LMR feature subset can eliminate redundant features in the row and column vectors in the air pressure feature space and supplement the missing information of the column and row vectors in each other. SE feature set, the optimal feature set, adds the number of features of different dimensions based on deleting redundant features, which helps improve the accuracy.

#### Principle of spearman maximal relevance (SMR)

(1) Calculate the ranks of all data in the preprocessed barometric pressure data. The elements in the barometric pressure feature matrix ${\varvec{Z}}$ are converted into two column vectors: ${\varvec{X}}$ and ${\varvec{Y}}$, respectively, corresponding to the sum of the elements ${x}_{i}$, ${y}_{i}(i\in \left\{\mathrm{0,1},...,N\right\})$ and are converted into the rankings in the respective column vectors in ascending order from smallest to largest, as and, which ultimately constitutes the sum of the respective corresponding column vectors ${\varvec{R}}({\varvec{X}})$, ${\varvec{R}}({\varvec{Y}})$.

The number of ranks of all data in the preprocessed barometric pressure data is calculated. The elements in the barometric pressure feature matrix are converted into two column vectors: ${\varvec{X}}$ and ${\varvec{Y}}$, respectively, corresponding to the sum of the elements, and converted into the ranks $R({x}_{i})$,$R({y}_{i})$ in the respective column vectors in ascending order from smallest to largest, as and, which ultimately constitutes the sum of the respective corresponding column vectors.

Where $N$ is the number of elements of the air pressure feature matrix for each sample, $N\le 120$.

(2) Calculate the correlation coefficient between the sums of the corresponding elements ${\varvec{R}}({\varvec{X}})$ and ${\varvec{R}}({\varvec{Y}})$ in two column vectors according to Eq. (5):

5 $$\rho = \frac{{\mathop \sum \nolimits_{i = 1}^{N} \left[ {R\left( {x_{i} } \right) - \overline{R\left( X \right)} } \right]\left[ {R\left( {y_{i} } \right) - \overline{R\left( Y \right)} } \right]}}{{\sqrt {\mathop \sum \nolimits_{i = 1}^{N} [R\left( {x_{i} } \right) - \overline{R\left( X \right)} ]^{2} \mathop \sum \nolimits_{i = 1}^{N} [R\left( {y_{i} } \right) - \overline{R\left( Y \right)} ]^{2} } }}$$

where, $\overline{R(X)}$ and $\overline{R(Y)}$ denote the mean values of rank $R(X)$ and $R(Y)$, $\overline{R(X)}=\frac{1}{N}{\sum }_{i=1}^{N}R({x}_{i})$, $\overline{R(Y)}=\frac{1}{N}{\sum }_{i=1}^{N}R({y}_{i})$ respectively. $N$ is the number of elements of the air pressure feature matrix for each sample, $N\le 120$.

From Eq. (5), it can be seen that the translation and scaling of ${\varvec{X}}$ and ${\varvec{Y}}$ does not affect the calculation of the correlation coefficient, so after normalization of ${\varvec{R}}({\varvec{X}})$ and ${\varvec{R}}({\varvec{Y}})$, the equation for the Spearman correlation coefficient can be transformed into:

6 $$\rho = Cov\left[ {R\left( {x_{i} } \right),R\left( {y_{j} } \right)} \right] = {\varvec{R}}\left( {\varvec{X}} \right){\varvec{R}}\left( {\varvec{Y}} \right)$$

In this way, in order to screen out the significant features with maximum rank correlation in each region without changing the rank characteristics after the standardization process of ${\varvec{R}}({\varvec{X}})$ and ${\varvec{R}}({\varvec{Y}})$, this paper converts the optimal solution of solving the objective function 1(8) under the original constraint 1(7) to the feature decomposition of the maximum rank correlation matrix (MRR matrix) ${\varvec{R}}({\varvec{X}}{)}^{{\varvec{T}}}{\varvec{R}}({\varvec{Y}}){\varvec{R}}({\varvec{Y}}{)}^{{\varvec{T}}}{\varvec{R}}({\varvec{X}})$ by constructing the Lagrangian function (9). The larger the eigenvalue is, the more suitable the corresponding eigenvector is as the solution vector of the optimization objective function, i.e., the number of significant features corresponding to each region.

Constraint 1 is:

7 $$s.t.\left\{ {\begin{array}{*{20}c} {{\varvec{\alpha}}_{1}^{{\varvec{T}}} {\varvec{\alpha}}_{1} = 1} \\ {{\varvec{\beta}}_{1}^{{\varvec{T}}} {\varvec{\beta}}_{1} = 1} \\ \end{array} } \right.$$

The objective function 1 is:

8 $$\max ({\varvec{\alpha}}_{1}^{{\varvec{T}}} {\varvec{R}}\left( {{\varvec{X}})^{{\varvec{T}}} {\varvec{R}}\left( {\varvec{Y}} \right){\varvec{\beta}}_{1} } \right)$$

Constructing a Lagrangian function:

9 $$L = {\varvec{\alpha}}_{1}^{{\varvec{T}}} {\varvec{R}}({\varvec{X}})^{{\varvec{T}}} {\varvec{R}}\left( {\varvec{Y}} \right){\varvec{\beta}}_{1} - \frac{\lambda }{2}\left( {{\varvec{\alpha}}_{1}^{{\varvec{T}}} {\varvec{\alpha}}_{1} - 1} \right) - \frac{\gamma }{2}\left( {{\varvec{\beta}}_{1}^{{\varvec{T}}} {\varvec{\beta}}_{1} - 1} \right)$$

Constraint 2 is:

10 $${\varvec{R}}({\varvec{X}})^{{\varvec{T}}} {\varvec{R}}\left( {\varvec{Y}} \right){\varvec{R}}({\varvec{Y}})^{{\varvec{T}}} {\varvec{R}}\left( {\varvec{X}} \right){\varvec{\alpha}}_{1} = \lambda^{2} {\varvec{\alpha}}_{1}$$

The objective function 2 is:

11 $$\max \lambda$$

In the process of constructing the salient feature subset ${\varvec{T}}$, the feature values and the corresponding feature vectors are sorted according to the cumulative contribution in ascending order, discarding the smaller feature values by setting a threshold, and the feature vectors corresponding to the screened feature values are utilized for feature extraction from ${\varvec{Z}}$. Then all the selected ${\boldsymbol{\alpha }}_{i}$ constitutes the load matrix ${\varvec{P}}=\left[{\alpha }_{1},{\alpha }_{2},...,{\alpha }_{i}\right]$. According to the load matrix ${\varvec{P}}$, the salient feature subset ${\varvec{T}}$ is obtained:

12 $${\mathbf{T}} = {\mathbf{ZP}} = \left[ {\begin{array}{*{20}c} {t_{0,0} } & {t_{0,1} } & \cdots & {t_{0,6} } \\ {t_{1,0} } & {t_{1,1} } & \cdots & {t_{1,6} } \\ \vdots & \vdots & \cdots & \vdots \\ {t_{8,0} } & {t_{8,1} } & \cdots & {t_{8,6} } \\ \end{array} } \right]$$

**Figure 8:**
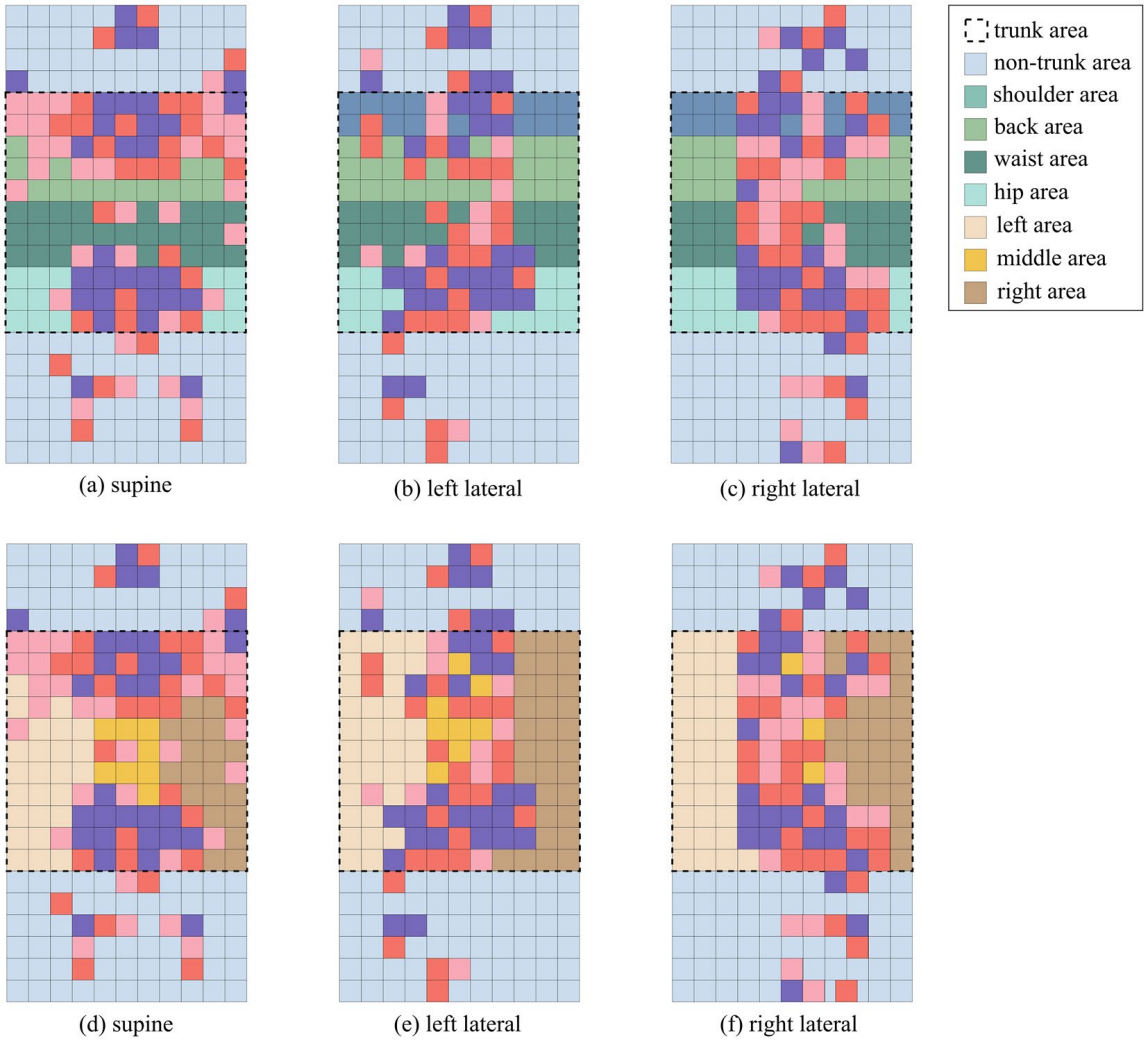
Illustrates a schematic diagram of pressure features in mattress divisions , wherein (**a**), (**b**), and (**c**) illustrate pressure features distributions for different sleep postures under horizontal division, and (**d**), (**e**), and (**f**) illustrate pressure feature distributions for different sleep postures under longitudinal division.

In optimizing the pressure features, the distribution of the salient feature subset in different sleep postures shows a certain regularity. For the longitudinal distribution, the pressure feature values are mainly along the spinal line; for the horizontal distribution, the pressure feature values are mainly in the four areas: shoulder, back, waist, and hips area. Therefore, Sub Sec. “Construction of SBWH (shoulder, back, waist, hip) feature subset” and “Construction of LMR (Left、Middle、Right) feature subset” will further investigate the feature sets mainly concentrated in the critical regions and construct the SBWH and LMR feature subsets according to the horizontal and longitudinal division strategies. Figure 8 demonstrates the distribution of the pressure eigenvalues on the mattress and the horizontal and longitudinal division.

#### Construction of SBWH (shoulder, back, waist, hip) feature subset

The process of constructing the SBWH feature subset is the process of reconstructing the salient feature subset ${\varvec{T}}$. According to the distribution of the human torso structure, the salient feature subset is laterally sliced into four parts: shoulder feature matrix ${{\varvec{T}}}_{0}$, back feature matrix ${{\varvec{T}}}_{1}$, waist feature matrix ${{\varvec{T}}}_{2}$, and hip feature matrix ${{\varvec{T}}}_{3}$. Finally, the horizontal boundary feature subset, filtered according to SMR, is reconstructed into a horizontal boundary feature subset together with the lateral boundary ratio feature subset obtained by feature enhancement. The horizontal boundary ratio feature subset is reconstructed as the SBWH feature subset.

For salient features ${t}_{i,j}$, the optimal features are selected by setting a threshold according to the structure of the human body and SMR. Thus, the optimal features are defined in the following equation:

13 $$t_{i,j} = \left\{ {\begin{array}{*{20}c} {\begin{array}{*{20}c} {t_{i,j} } & {t_{i,j} \ge R_{Thre} } \\ \end{array} } \\ {\begin{array}{*{20}c} 0 & {{\text{otherwise}}} \\ \end{array} } \\ \end{array} } \right.$$

14 $$R_{Thre} = \frac{3}{5}{\text{min}}\left\{ {M_{R} } \right\}$$

where ${M}_{R}$ is the set of maximum values in each row in each horizontal area.

Sixteen horizontal boundary features are screened out from the salient feature subset ${\varvec{T}}$, each area obtains a $1\times 4$ horizontal boundary feature vector ${{\varvec{a}}}_{{\varvec{i}}}(i\in \{\mathrm{0,1},\mathrm{2,3}\})$, constituting a horizontal boundary matrix ${\varvec{A}}$.

15 $${\varvec{A}} = \left[ {\begin{array}{*{20}c} {{\varvec{a}}_{0} } & {{\varvec{a}}_{1} } & {{\varvec{a}}_{{2\user2{ }}} \user2{ a}_{3} } \\ \end{array} } \right]^{{\varvec{T}}}$$

Subsequently, feature enhancement is performed: the corresponding elements between any two row vectors ${{\varvec{a}}}_{{\varvec{i}}}$ are transformed according to the formula of the ratio vector to obtain six horizontal ratio eigenvectors ${{\varvec{b}}}_{{\varvec{i}}}(i\in \{\mathrm{0,1},\mathrm{2,3}\})$, constituting a horizontal ratio feature matrix ${\varvec{B}}$. The ratio transformation is defined as follows:

16 $$Rat(m_{i} ,n_{j} ) = \left[ {m_{i,0} /n_{j,0} ,m_{i,1} /n_{j,1} ,...,m_{i,k} /n_{j,k} } \right]$$

where ${{\varvec{m}}}_{{\varvec{i}}},{{\varvec{n}}}_{{\varvec{j}}}$ is a $1\times k$ vector.

Thus, the horizontal ratio feature matrix ${\varvec{B}}$ is obtained:

17 $$B = \left[ {\begin{array}{*{20}c} {{\varvec{b}}_{0} } & {{\varvec{b}}_{1} } & {{\varvec{b}}_{2} } & {{\varvec{b}}_{3} } & {{\varvec{b}}_{4} } & {{\varvec{b}}_{5} } \\ \end{array} } \right]^{T}$$

where the shoulder-to-back ratio eigenvector ${{\varvec{b}}}_{0}=\mathit{Rat}({{\varvec{a}}}_{0},{{\varvec{a}}}_{1})$, shoulder-to-waist ratio eigenvector ${{\varvec{b}}}_{1}=\mathit{Rat}({{\varvec{a}}}_{0},{{\varvec{a}}}_{2})$, shoulder-to-hip ratio eigenvector ${{\varvec{b}}}_{2}=\mathit{Rat}({{\varvec{a}}}_{0},{{\varvec{a}}}_{3})$, back-to-waist ratio eigenvector ${{\varvec{b}}}_{3}=\mathit{Rat}({{\varvec{a}}}_{1},{{\varvec{a}}}_{2})$, back-to-hip ratio eigenvector ${{\varvec{b}}}_{4}=\mathit{Rat}({{\varvec{a}}}_{1},{{\varvec{a}}}_{3})$, and waist-to-hip ratio eigenvector ${{\varvec{b}}}_{5}=\mathit{Rat}({{\varvec{a}}}_{2},{{\varvec{a}}}_{3})$ respectively. Finally, the horizontal boundary feature vectors and the horizontal ratio feature vectors are combined to form the SBWH feature subset.

#### Construction of LMR (Left、Middle、Right) feature subset

In constructing the LMR feature subset, the salient feature subset ${\varvec{T}}$ was longitudinally divided into three parts according to the different sleep postures: the left-pressure feature matrix ${\boldsymbol{\rm T}}_{4}$, the middle-pressure feature matrix ${\boldsymbol{\rm T}}_{5}$, and the right-pressure feature matrix ${{\varvec{T}}}_{6}$. Finally, the longitudinal boundary features subset filtered according to SMR was combined with the longitudinal ratio feature subset obtained by feature enhancement to reconstruct the LMR feature subset.

For the number of salient features ${t}_{i,j}$ in the three longitudinal areas, the optimal features are selected by setting a threshold based on the difference in sleep postures and SMR:

18 $$t_{i,j} = \left\{ {\begin{array}{*{20}c} {\begin{array}{*{20}c} {t_{i,j} } & {t_{i,j} \ge C_{Thre} } \\ \end{array} } \\ {\begin{array}{*{20}c} 0 & {{\text{otherwise}}} \\ \end{array} } \\ \end{array} } \right.$$

19 $$C_{Thre} = \frac{3}{5}{\text{min}}\left\{ {M_{C} } \right\}$$

where ${M}_{C}$ is the set of maximum values in each column in each vertical region.

Finally, 18 longitudinal boundary features are selected from the salient feature subset ${\varvec{T}}$. They constitute three longitudinal boundary feature vectors ${{\varvec{c}}}_{{\varvec{i}}}(i\in \{\mathrm{0,1},2\})$ of each of ${\boldsymbol{\rm T}}_{4}$, ${\boldsymbol{\rm T}}_{5}$, and ${{\varvec{T}}}_{6}$, thus, realizing the feature filtering.

20 $${\varvec{C}} = \left[ {\begin{array}{*{20}c} {{\varvec{c}}_{0} } & {{\varvec{c}}_{1} } & {{\varvec{c}}_{2} } \\ \end{array} } \right]$$

where ${{\varvec{c}}}_{{\varvec{i}}}$ is the eigenvector of the $i{\text{th}}$ column in the longitudinal boundary matrix ${\varvec{C}}$. Subsequently, according to the formula of the ratio vector, we transform the corresponding elements of the two vectors between longitudinal boundary eigenvectors ${{\varvec{c}}}_{0}$, ${{\varvec{c}}}_{1}$ and ${{\varvec{c}}}_{2}$. Three $6\times 1$ longitudinal ratio eigenvectors ${{\varvec{d}}}_{{\varvec{i}}}(i\in \{\mathrm{0,1},2\})$ are obtained by feature enhancement, forming a longitudinal ratio feature matrix ${\varvec{D}}$:

21 $${\varvec{D}} = \left[ {\begin{array}{*{20}c} {{\varvec{d}}_{0} } & {{\varvec{d}}_{1} } & {{\varvec{d}}_{2} } \\ \end{array} } \right]$$

where the left-middle ratio eigenvector ${{\varvec{d}}}_{0}=\mathit{Rat}({{{\varvec{c}}}_{0}}^{T},{{{\varvec{c}}}_{1}}^{T}{)}^{T}$, the left–right ratio eigenvector ${{\varvec{d}}}_{1}=\mathit{Rat}({{{\varvec{c}}}_{0}}^{T},{{{\varvec{c}}}_{2}}^{T}{)}^{T}$, and the right-middle ratio eigenvector ${{\varvec{d}}}_{2}=\mathit{Rat}({{{\varvec{c}}}_{2}}^{T},{{{\varvec{c}}}_{1}}^{T}{)}^{T}$. Finally, the longitudinal boundary feature vectors are combined with the ratio feature vectors to form the LMR(Left, Middle, right) feature subset.

Finally, the SBWH feature subset is fused with the LMR feature subset to obtain the final SE features used as inputs to the classification model. Such fusion reduces the redundant information in the air pressure values, i.e., the air cushion not squeezed by different sleep postures during sleep activities, to optimize the computation and feature space. The SE feature set adds features of different dimensions, which facilitates the construction of a stable classification model.

### Ensemble classifier

AdaBoost algorithm is essentially the process of sleeper recognition and classification by iterating the error rate of SE feature samples’ weights, and if the number of times an SE feature sample is misclassified increases, the weights will also increase. SVM classifier algorithms are especially suitable for the classification of small samples, and the combination of AdaBoost algorithms and SVM algorithms increases the requirement for the generalization performance of strong classifiers. The AdaBoost algorithm improves the basic performance of the classical SVM classifier with sleeping classification accuracy and can screen the optimal classification kernel parameters for the AdaBoost-SVM classifier, so as to obtain multiple weak classifiers, which can be trained into strong classifiers after iteration, integration, and judgment of the training error rate. The weak classifier based on SVM ensures the variability of each round of screening and training, but the proportionality of sample weights should be considered in the classification and selection of samples in order to improve the efficiency of training and reduce the training time.

**Figure 9:**
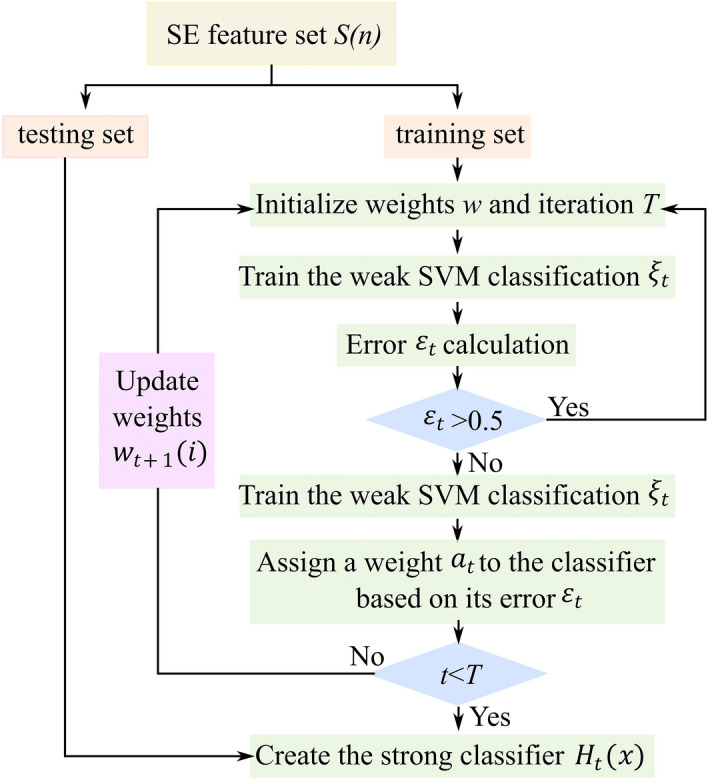
Flowchart of AdaBoost-SVM model.

Figure 9 presents the AdaBoost-SVM model flowchart, where each weak classifier structure in the AdaBoost-SVM model is used to generate strong classifiers after several iterations. The set of samples with all SE features is fed into the AdaBoost-SVM classifier and the weight values of the sample data are initialized. Set the number of iterations, if during the iteration process, it is found that the positive samples are not completely recognized can temporarily increase the number of iterations. Calculate the error rate of each weak classifier on the sample training set separately, and if the error is greater than 0.5, update the sample weights of the original SE features so that the misclassified samples receive more attention in subsequent iterations. Finally, strong classifiers that can be used for sleep posture recognition are generated.

where $S(n)=\{({x}_{1},{y}_{1}),({x}_{2},{y}_{2}),...,({x}_{i},{y}_{i})\}$, represents $N$ samples, ${x}_{i}$ represents the ith sample of the SE feature set, and ${y}_{i}$ represents the type of sleep posture to which the ith sample belongs. $\upomega$ is the weight of the sample, $T$ is the total number of iterations for model training, and is the number of iterations carried out. ${\upxi }_{t}$ is the RBF kernel SVM classifier. ${\upvarepsilon }_{{\text{t}}}$ is the error rate of the classifier ${\upxi }_{t}$. ${w}_{t+1}(i)$ is the weight of the training set of the weight of the ith sample, and ${a}_{t}$ is the weight of the classifier ${\upxi }_{t}$. ${H}_{t}(x)$ is the final strong classifier.

22 $$\varepsilon_{t} = \mathop \sum \limits_{i = 1}^{N} \omega_{i} ,\,y_{i} \ne \xi_{t} \left( {x_{i} } \right)$$

23 $$a_{t} = \frac{1}{2}\log \frac{{1 - \varepsilon_{t} }}{{\varepsilon_{t} }}$$

24 $${\text{w}}_{t + 1} \left( i \right) = \frac{{{\text{w}}_{t} \left( i \right)exp\left( { - a_{t} y_{i} \xi_{t} \left( {x_{i} } \right)} \right)}}{{C_{t} }}$$

25 $$H_{t} \left( x \right) = sign\left( {\mathop \sum \limits_{t = 1}^{T} a_{t} h_{t} \left( x \right)} \right)$$

where $i=\mathrm{1,2},3,\dots ,N$*.* Where ${C}_{t}$ is a normalization factor satisfying $\sum_{i=1}^{N}{w}_{t+1}\left(i\right)=1$**.**

In the actual process of sleep posture recognition, noise and outliers may be introduced due to the process of feature extraction in the horizontal and vertical divisions, especially in different parts of the mattress due to movement and other factors that generate errors; certain sleep postures may appear more frequently than others, resulting in inter-sample imbalance in the data; and features extracted in the horizontal and vertical divisions present complex nonlinear relationships. The AdaBoost-SVM algorithm’s iterative process identifies and reduces the negative impact of these noises and outliers on the classification performance, improves the classification performance on the unbalanced dataset, and handles these nonlinear features effectively.

## Experiments and results

All experiments were run on a standard desktop computer with 8 GB RAM and an Intel i7-3070 CPU using the Anaconda platform, with the programming language Python 3.8.5. Twenty-six subjects (13 males and 13 females) participated in the sleep posture recognition experiment. The age of the participants ranged from 20 to 26, height ranged from 170 to 186, and weight ranged from 50 to 90 kg.

Each test subject recorded 40 samples during the data collection, including 20 supine and lateral postures. The proposed model was tested using k-fold cross-validation ($k={10}$) and Leave One Out Cross-validation (LOOCV). In tenfold cross-validation, we divide the data into ten subsets, of which 10% is used for testing and 90% for training. It is repeated ten times, and finally, the average value is calculated as the accuracy of sleep posture recognition.

### Validation of SMR feature extraction strategy

In order to quantify the performance of SMR in feature extraction for sleep posture recognition, we take the raw pressure data, significant feature subset, SBWH feature subset, LMR feature subset, and SE feature set as inputs to the AdaBoost classifier, respectively. The recognition accuracies in the five cases are compared using tenfold cross-validation and LOOCV.

Table 1 shows the average accuracy under different stages of data as input in feature extraction. The accuracy of the classifier increases with the critical features in the feature set, where the accuracy of the original data is 0.886 and that of the SE feature set reaches 0.997 since SMR can eliminate redundant information and add strong discriminative vital features. When SWBH and LMR feature subsets are used as inputs, the difference in accuracy between supine and lateral postures is not significant because, although these have different division criteria and discriminative features, their intersection is valid. It indicates that the strong SMR feature extraction capability enables the classifier to better capture the relationship between feature inputs and sleep postures.

**Table 1:** Sleep posture recognition precision with different Sleep posture feature data.

Validation Scheme	tenfold		LOOCV	
Posture	Supine	Lateral	Supine	Lateral
Raw data	0.889	0.884	0.813	0.806
Salient feature subset	0.910	0.906	0.867	0.854
SBWH feature subset	0.965	0.954	0.916	0.905
LMR feature subset	0.946	0.968	0.901	0.915
SE feature set	0.996	**0.998**	**0.991**	0.989

Significants values are in bold.

### Comparison of traditional base classification models

In order to compare the performance of the SPR-DE on sleep posture recognition, we compare SPR-DE with traditional base classification models (SVM, Naive Bayes, KNN, Decision Tree), and the SE feature set is directly used as an input to the traditional classification model as well as SPR-DE. Both tenfold cross-validation and LOOCV were used for each combination of cases to obtain the average accuracy of sleep posture recognition corresponding to each case.

Table 2 demonstrates the average accuracies of the traditional base classifier and the SPR-DE under different validation methods. The highest accuracy of SPR-DE under both tenfold and LOOCV is because SPR-DE is a sleep posture recognition model focusing on the combination of feature extraction strategy and integrated algorithm training. the AdaBoost-SVM algorithm can consider the weights of each base classifier fully. Under LOOCV, when faced with the test set data not involved in training, the accuracy of traditional base classifiers decreases significantly. At the same time, the SPR-DE still performs robustly and achieves an accuracy of 0.996 and 0.991, with the slightest change of 0.7% in the accuracy, which indicates that the features and AdaBoost–SVM classifiers, increasing the classification credibility.

**Table 2:** Sleep posture recognition accuracy of different baseline models with SE feature set.

Validation Scheme	tenfold		LOOCV	
Posture	Supine	Lateral	Supine	Lateral
SVM	0.968	0.968	0.939	0.933
Naive Bayes	0.971	0.969	0.917	0.907
kNN(k = 12)	0.964	0.960	0.942	0.924
Decision Tree	0.970	0.964	0.943	0.939
SPR-DE	0.996	**0.998**	**0.996**	0.991

Significants values are in bold.

Table 3 and 4 show the performance of different classification models on sleep posture recognition under tenfold and LOOCV validation methods, including four performance metrics: Accuracy, Recall, F1-score, and MCC (Mattews Correlation Coefficient).

**Table 3:** Evaluation indexes of different base models under the LOOCV validation scheme.

Models	SVM	Naive Bayes	kNN	Decision Tree	SPR-DE
Accuracy	0.936	0.912	0.933	0.941	**0.989**
Recall	0.923	0.935	0.927	0.932	**0.973**
F1_score	0.936	0.913	0.933	0.940	**0.989**
MCC	0.925	0.935	0.926	0.929	**0.973**

Significants values are in bold.

**Table 4:** Evaluation indexes of different base models under the tenfold validation scheme.

Models	SVM	Naive Bayes	kNN	Decision Tree	SPR-DE
Accuracy	0.968	0.970	0.962	0.967	**0.999**
Recall	0.947	0.944	0.935	0.941	**0.989**
F1_score	0.968	0.969	0.962	0.967	**0.998**
MCC	0.947	0.944	0.934	0.941	**0.990**

Significants values are in bold.

Table 3 demonstrates that the classification algorithm adopted by SPR-DE has the best performance in terms of precision, F1-score, recall, and MCC under the LOOCV validation method, with the precision reaching 0.989, which is an improvement of 0.045–0.077 compared to the other classifiers, and the F1-score reaching 0.989, which is an improvement of 0.049–0.076. This is because in the case of data diversity being large, when only one sample is not enough to represent the distribution of the whole dataset, the model is not only required to have a high recognition accuracy but also requires a strong generalization ability and is not perturbed by outliers. The feature extraction strategy as well as the integrated classifier in SPR-DE can enhance the robustness of the model by focusing on the main support regions of the human body and the amount of spine features.

Table 4 shows that the classification algorithm used in SPR-DE can also perform the best in terms of precision, F1-score, recall, and MCC under the tenfold validation method. The precision reaches 0.999, which is improved by 0.029–0.037 compared to other classifiers; the F1-score reaches 0.998, which is improved by 0.029–0.036. SPR-DE not only maintains a high precision of sleep recognition, but also has a strong correlation between the model’s prediction and the actual result, and the overall performance of the model is excellent.

**Figure 10:**
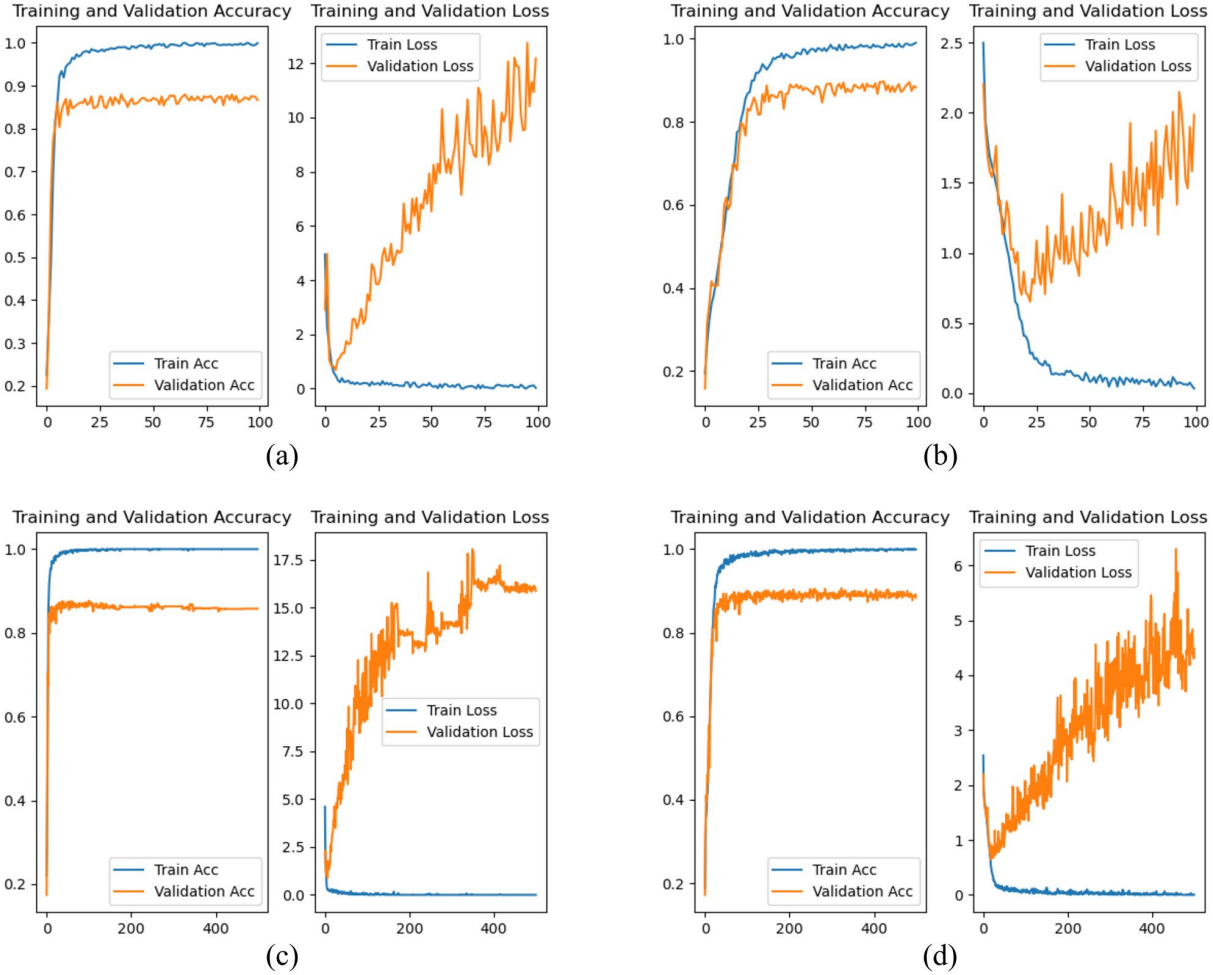
Relationship between the classification accuracy rate and the number of iterations of the SPR-DE model under two validation modes, where (**a**) and (**b**) are the results of 100 iterations under the tenfold validation mode and LOOCV validation mode, respectively, and (**c**) and (**d**) are the results of 500 iterations under the tenfold validation mode and LOOCV validation mode, respectively.

Figure 10 shows the relationship between the loss function value and the number of iterations for the test and training sets under the two validation methods. SPR-DE can converge quickly and reach a steady state under either validation method because SMR provides critical features for the classification algorithm. In LOOCV, the SPR-DE error is relatively higher, and the loss function value oscillates significantly because some data from the test set are not included in the training set.

### Comparison of different ensemble learning models

For the sleep posture recognition task based on ensemble barometric data adopted in this paper, the SPR-DE is compared with other ensemble classifiers (Random Forest, GBDT, Bagging, XGBoost). Each is evaluated using tenfold and LOOCV to get the average accuracy of each model.

As can be seen from Table 5, SPR-DE outperforms other ensemble classifiers and achieves better results, both in tenfold and LOOCV. Under tenfold, SPR-DE recognition accuracy reaches 0.998, which is the 3.7% improvement compared to other classifiers, and under LOOCV, the accuracy of each ensemble classifier decreases significantly. The performance of our proposed SPR-DE remains robust because the combination of SMR and AdaBoost-SVM can consider both feature space and model integration, which improves the generalization ability and performance of the final model.

**Table 5:** Sleep posture recognition precision of different ensemble classifiers with SE feature set.

Validation Scheme	tenfold		LOOCV	
Posture	Supine	Lateral	Supine	Lateral
RandomForest	0.960	0.961	0.906	0.915
Bagging	0.973	0.979	0.851	0.857
GDBT	0.974	0.977	0.849	0.851
XGBoost	0.974	0.977	0.835	0.840
SPR-DE	0.996	**0.998**	**0.996**	0.991

Significants values are in bold.

## Discussion

Table 6 shows the results of comparing the SPR-DE model proposed in this paper with our similar state-of-the-art works. We summarize the Sensor type, Data type, Preprocessing techniques, Recognition Algorithm, Evaluation measures, Accuracy rate, and R&A (Recognize sleep postures and Adjust hardness according to the sleep postures at the same time) which summarize the advantages and disadvantages of the method proposed in this paper with other pressure sensor based mattresses.

**Table 6:** Comparison between this work and recent published references.

Methods	Sensor and Data		technique		System specification		
	Sensor type	Data type	Preprocessing techniques	Recognitionalgorithm	Evaluation measures	Accuracy	R&A simultaneously
Proposed method	barometric sensor	air pressure	Threshold Filtering + Neighborhood filtering	SMR + AdaBoost-SVM	Accuracy F1-score	99.9%	yes
^35^	Pressure sensitive sheet	pressure image	CNN with Transfer Learning		Accuracy Confusion matrix	91.24%	no
^34^	FSR sensor	pressure image	HOG + LBP	FFANN	Accuracy Confusion matrix	97%	no
^45^	FSR sensor	pressure image	No feature extraction	Deep neural network	Accuracy	99.7%	no
^44^	FSR sensor	pressure image	spatio-temporal median filter	CNN	Accuracy	99.8%	no
^46^	FSR sensor	pressure image	Fuzzy representation	CNN	precision, recall, F1-score and accuracy	98.2%	NO
^39^	FSR sensor	pressure data	No feature extraction	Neural Network Bayesian Network	accuracy	97.1%	NO

In the above methods, although all the sensors collect all the pressure signals^34,35,39,44–46^, in the algorithm processing part, still all the pressure data are converted to pressure image data as the classifier input, which is high in accuracy but has a large computational cost and a large number of sensors, whereas the SPR-DE model adopts pressure data as the input of the classifier, not pressure image data, which does not need to go through a complex image processing algorithms, which speeds up the processing speed; the pressure data exists in the form of a numerical matrix, and its data volume is small compared to the image data. This means that fewer computational resources are needed to process the pressure data, which can reduce the hardware requirements for running the model, especially important for the real-time sleep posture recognition system. Although pressure data is used as the classifier input in^39^, it mainly measures pressure changes under the chest, which cannot understand the pressure tolerance of different regions and body parts, and cannot provide suggestions to enhance sleep comfort. The non-image data features related to sleep postures in the SPR-DE model make it possible to design a sleep posture recognition system that takes into account the R&A of mattress comfort and sleep posture recognition. becomes possible.

On the other hand, reducing the complexity of the embedded system while improving the accuracy of sleep posture recognition, such as^35^ in although less number of sensors used, but the average accuracy of 91.2%, in the accuracy of sleep posture recognition may not meet the requirements of the engineering applications of R&A accurate recognition of the sleep posture, compared to this paper’s proposed SPR-DE model in the processing of pressure data with an accuracy of 99.9%; From exploring the performance of aptitude sensors combined with CNN, the accuracy of sleep posture recognition reaches 98.2%^46^, but a flexible pressure sensor pad is used, which is easily affected by various disturbing factors in the sleeping environment, such as the movement of the bed, different bed materials or changes in the bed sheet, and is unable to work stably under the changing environmental conditions, which may not meet the robustness requirements for R&A to accurately recognize the sleep posture.

The SMR feature extraction strategy in SPR-DE, which focuses on the shoulder-hip and spine feature quantities, can capture the pressure distribution differences caused by different sleep postures in a more detailed way, and this segmentation improves the sensitivity of the model to subtle changes, which in turn improves the accuracy; while AdaBoost-SVM learns the complex features in different divisions step by step and uses the efficient classification capability of SVM in nonlinear features as well as boundary optimization, which makes it possible for R&A to accurately recognize sleep postures. Boundary optimization of efficient classification ability, so that the model can automatically adjust according to the actual distribution of data, to provide personalized support for different sleep postures, and to improve the accuracy and generalization performance of sleep posture recognition.

## Conclusion and future work

The sleep posture recognition model-SPR-DE proposed in this study for airbed structure, analyzes 121 pressure data of the human body’s trunk region through SMR feature extraction register and horizontal and vertical division, instead of pressure image data as model input, focusing on shoulder-hip feature volume and spine features, directly captures the closely related changes in sleep posture, provides more targeted and yet graded information, and enhances the model’s sensitivity to the sensitivity to differences in sleep postures. The results show that the accuracy of SPR-DE can reach 0.997. Compared with other models, the accuracy was improved by 2.9–7.7% and F1-score by 0.029 to 0.076. This method can utilize the trunk area for sleep posture recognition while ensuring sufficient information with high recognition accuracy, reducing the number of sensors and, at the same time, reducing computational pressure brought about by the processing of the pressure data, which will contribute to the improvement of sleep posture recognition system with the low production and computational cost to provide ideas for portability.

In the future, we will study how the mattress conditioning system can use key features of the sleep posture recognition model to accurately understand which areas are under more pressure, to provide different areas with body profiles and preferences that match the individual, and to explore the provision of a more comfortable and healthy personalized sleep experience for different users.

## Data Availability

The data supporting the findings of this study are available from the corresponding author upon reasonable request.

## References

[1] Matricciani L (2018). Rethinking the sleep-health link. Sleep Health.

[2] Liew SC, Aung T (2021). Sleep deprivation and its association with diseases-a review. Sleep Med..

[3] Office of Disease Prevention and Health Promotion, “Sleep Health,” 2021. [Online]. Available: https://www.healthypeople.gov/2020/topics-objectives/topic/sleep-health/national-snapshot

[4] The SUN, “DREAM TEAM People are Arguing About What is the Best Position to Sleep in But Which One Are You?” 2020.[Online].Available: https://www.thesun.co.uk/fabulous/10791045/sleep-position-best-people-argue-night/

[5] Thoracic and Sleep Group Queensland, “What is the Best Position to Sleep in?” 2021. [Online]. Available: http://thoracicandsleep.com.au/blog/what-is-the-best-position-to-sleep-in/

[6] Frange C, Coelho FMS (2022). Sleep Medicine and Physical Therapy: A Comprehensive Guide for Practitioners.

[7] Hong TT-H, Wang Y, Wong DW-C, Zhang G, Tan Q, Chen TL-W, Zhang M (2022). The influence of mattress stiffness on spinal curvature and intervertebral disc stress—An experimental and computational study. Biology.

[8] SAE-LEE, W. I. T. T. H. A. W. I. N., & Intolo, P. Innovative lumbo-pelvic seating cushion to improve lumbo-pelvic posture during sitting in office worker (Doctoral dissertation, Srinakharinwirot University), (2021).

[9] Gianfilippo C, Talesa GR, Giuseppe T, Eugenio J, Gaetano M, Leonardo P (2021). What type of mattress should be chosen to avoid back pain and improve sleep quality? Review of the literature. J. Orthop. Traumatol..

[10] Yu-Chi L, Chih-Yun L, Mao-Jiun W (2020). Better combination of thickness and hardness of mattress topper for supine sleeping posture: A physiological measurements evaluation. Int. J. Ind. Ergon..

[11] Fang JJ, Shen LM (2023). Analysis of sagittal spinal alignment at the adolescent age: For furniture design. Ergonomics.

[12] Norasi H, Tetteh E, Sarker P, Mirka GA, Hallbeck MS (2022). Exploring the relationship between neck flexion and neck problems in occupational populations: A systematic review of the literature. Ergonomics.

[13] Chao Y, Liu T, Shen LM (2023). Method of recognizing sleep postures based on air pressure sensor and convolutional neural network: For an air spring mattress. Eng. Appl. Artif. Intell..

[14] Chao Y, Shen LM (2022). Nonlinear stiffness characteristics and model of air spring for mattress based on finite element and numerical analysis. Adv. Theory Simul..

[15] Chao Y, Shen LM, Liu MP (2021). Mechanical characteristic and analytical model of novel air spring for ergonomic mattress. Mech. Ind..

[16] Hussain Z, Sheng QZ, Zhang WE, Ortiz J, Pouriyeh S (2022). Non-invasive techniques for monitoring different aspects of sleep: A comprehensive review. ACM Trans. Comput. Healthc. (HEALTH).

[17] De Zambotti M, Cellini N, Goldstone A, Colrain IM, Baker FC (2019). Wearable sleep technology in clinical and research settings. Med. Sci. Sports Exerc..

[18] Jeon S, Park T, Paul A, Lee YS, Son SH (2019). A wearable sleep position tracking system based on dynamic state transition framework. IEEE Access.

[19] Roshini A, Kiran KVD (2022). An enhanced posture prediction-Bayesian network algorithm for sleep posture recognition in wireless body area networks. Int. J. Telemed. Appl..

[20] Schätz M, Procházka A, Kuchyňka J, Vyšata O (2020). Sleep apnea detection with polysomnography and depth sensors. Sensors.

[21] Alinia P, Samadani A, Milosevic M, Ghasemzadeh H, Parvaneh S (2020). Pervasive lying posture tracking. Sensors.

[22] Tam AYC, So BPH, Chan TTC, Cheung AKY, Wong DWC, Cheung JCW (2021). A blanket accommodative sleep posture classification system using an infrared depth camera: A deep learning approach with synthetic augmentation of blanket conditions. Sensors.

[23] Luo B, Yang Z, Chu P, Zhou J (2023). Human sleep posture recognition method based on interactive learning of ultra-long short-term information. IEEE Sens. J..

[24] Kiriazi JE, Islam SMM, Boric-Lubecke O, Lubecke VM (2021). Sleep posture recognition with a dual-frequency cardiopulmonary Doppler radar. IEEE Access.

[25] Islam SMM, Lubecke VM (2022). Sleep posture recognition with a dual-frequency microwave Doppler radar and machine learning classifiers. IEEE Sens. Lett..

[26] Lai DK-H (2023). Dual ultra-wideband (UWB) radar-based sleep posture recognition system: Towards ubiquitous sleep monitoring. Eng. Regen..

[27] Zheng Z, Zhang D, Liang X, Liu X, Fang G (2023). Unsupervised human contour extraction from through-wall radar images using dual UNet. IEEE Geosci. Remote Sens. Lett..

[28] Yue S, Yang Y, Wang H, Rahul H, Katabi D (2020). BodyCompass: Monitoring sleep posture with wireless signals. Proc. ACM Interact. Mob. Wearable Ubiquitous Technol..

[29] Rasouli D, M. S., & Payandeh, S. (2019). A novel depth image analysis for sleep posture estimation. J. Ambient Intell. Humaniz. Comput..

[30] Zhai B, Perez-Pozuelo I, Clifton EA, Palotti J, Guan Y (2020). Making sense of sleep: Multimodal sleep stage classification in a large, diverse population using movement and cardiac sensing. Proc. ACM Interact. Mobile Wearable Ubiquitous Technol..

[31] Liu J, Wu D, Wang Z, Jin X, Dong F, Jiang L, Cai C (2020). Automatic sleep staging algorithm based on random forest and hidden Markov model. Comput. Model. Eng. Sci..

[32] Heydarzadeh M, Nourani M, Ostadabbas S, Heydarzadeh M, Nourani M, Ostadabbas S (2016). In-bed posture classification using deep autoencoders. 2016 38th Annual International Conference of the IEEE Engineering in Medicine and Biology Society (EMBC).

[33] Kim TH, Kwon SJ, Choi HM, Hong YS (2019). Determination of lying posture through recognition of multitier body parts. Wirel. Commun. Mob. Comput..

[34] Matar G, Lina JM, Kaddoum G (2019). Artificial neural network for in-bed posture classification using bed-sheet pressure sensors. IEEE J Biomed Health Inf..

[35] Hu Q, Tang X, Tang W (2020). A real-time patient-specific sleep posture recognition system using pressure sensitive conductive sheet and transfer learning. IEEE Sens. J..

[36] Kau LJ, Wang MY, Zhou H (2023). Pressure-sensor-based sleep status and quality evaluation system. IEEE Sens. J..

[37] Diao H, Chen C, Chen W, Yuan W, Amara A, Diao H, Chen C, Chen W, Yuan W, Amara A (2021). Unobtrusive smart mat system for sleep posture recognition. 2021 IEEE International Symposium on Circuits and Systems (ISCAS).

[38] Li YY, Wang SJ, Hung YP (2022). A vision-based system for in-sleep upper-body and head pose classification. Sensors.

[39] Viriyavit W, Sornlertlamvanich V (2020). Bed position classification by a neural network and bayesian network using noninvasive sensors for fall prevention. J. Sens..

[40] Tang K, Kumar A, Nadeem M, Maaz I (2021). CNN-based smart sleep posture recognition system. IoT.

[41] Wang ZW, Wang SK, Wan BT, Song WW (1892). A novel multi-label classification algorithm based on K-nearest neighbor and random walk. Int. J. Distrib. Sens. Netw..

[42] Zhao A, Dong J, Zhou H (2020). Self-supervised learning from multi-sensor data for sleep recognition. IEEE Access.

[43] Byeon YH, Lee JY, Kim DH, Kwak KC (2020). Posture recognition using ensemble deep models under various home environments. Appl. Sci..

[44] Davoodnia V, Etemad A, Davoodnia V, Etemad A (2019). Identity and posture recognition in smart beds with deep multitask learning. 2019 IEEE International Conference on Systems, Man and Cybernetics (SMC).

[45] Enokibori Y, Mase K (2018). Data augmentation to build high performance DNN for in-bed posture classification. J. Inf. Process..

[46] Rodríguez AP, Gil D, Nugent C, Quero JM, Rodríguez AP, Gil D, Nugent C, Quero JM (2020). In-bed posture classification from pressure mat sensors for the prevention of pressure ulcers using convolutional neural networks. Bioinformatics and Biomedical Engineering: 8th International Work-Conference, IWBBIO 2020, Granada, Spain, May 6–8, 2020, Proceedings 8.

